# Optimized Left Ventricular Endocardial Stimulation Is Superior to Optimized Epicardial Stimulation in Ischemic Patients With Poor Response to Cardiac Resynchronization Therapy: A Combined Magnetic Resonance Imaging, Electroanatomic Contact Mapping, and Hemodynamic Study to Target Endocardial Lead Placement

**DOI:** 10.1016/j.jacep.2016.04.006

**Published:** 2016-12

**Authors:** Jonathan M. Behar, Tom Jackson, Eoin Hyde, Simon Claridge, Jaswinder Gill, Julian Bostock, Manav Sohal, Bradley Porter, Mark O'Neill, Reza Razavi, Steve Niederer, Christopher Aldo Rinaldi

**Affiliations:** Department of Imaging Sciences and Biomedical Engineering, King’s College London & Guy’s and St Thomas’ Hospital, London, United Kingdom

**Keywords:** cardiac magnetic resonance imaging, CRT, electroanatomic map, endocardial pacing, AHR, acute hemodynamic response, CMR, cardiac magnetic resonance, CRT, cardiac resynchronization therapy, EAM, electroanatomic mapping, LV, left ventricle/ventricular, LVendo, left ventricular endocardium, LVepi, optimal epicardial response, LVepi1, implanted LV lead, LVepi2, temporary LV lead, Q-LV, first ventricular depolarization

## Abstract

**Objectives:**

The purpose of this study was to identify the optimal pacing site for the left ventricular (LV) lead in ischemic patients with poor response to cardiac resynchronization therapy (CRT).

**Background:**

LV endocardial pacing may offer benefit over conventional CRT in ischemic patients.

**Methods:**

We performed cardiac magnetic resonance, invasive electroanatomic mapping (EAM), and measured the acute hemodynamic response (AHR) in patients with existing CRT systems.

**Results:**

In all, 135 epicardial and endocardial pacing sites were tested in 8 patients. Endocardial pacing was superior to epicardial pacing with respect to mean AHR (% change in dP/dt_max_ vs. baseline) (11.81 [-7.2 to 44.6] vs. 6.55 [-11.0 to 19.7]; p = 0.025). This was associated with a similar first ventricular depolarization (Q-LV) (75 ms [13 to 161 ms] vs. 75 ms [25 to 129 ms]; p = 0.354), shorter stimulation–QRS duration (15 ms [7 to 43 ms] vs. 19 ms [5 to 66 ms]; p = 0.010) and shorter paced QRS duration (149 ms [95 to 218 ms] vs. 171 ms [120 to 235 ms]; p < 0.001). The mean best achievable AHR was higher with endocardial pacing (25.64 ± 14.74% vs. 12.64 ± 6.76%; p = 0.044). Furthermore, AHR was significantly greater pacing the same site endocardially versus epicardially (15.2 ± 10.7% vs. 7.6 ± 6.3%; p = 0.014) with a shorter paced QRS duration (137 ± 22 ms vs. 166 ± 30 ms; p < 0.001) despite a similar Q-LV (70 ± 38 ms vs. 79 ± 34 ms; p = 0.512). Lack of capture due to areas of scar (corroborated by EAM and cardiac magnetic resonance) was associated with a poor AHR.

**Conclusions:**

In ischemic patients with poor CRT response, biventricular endocardial pacing is superior to epicardial pacing. This may reflect accessibility to sites that cannot be reached via coronary sinus anatomy and/or by access to more rapidly conducting tissue. Furthermore, guidance to the optimal LV pacing site may be aided by modalities such as cardiac magnetic resonance to target delayed activating sites while avoiding scar.

## Graphical abstract

**Figure:**
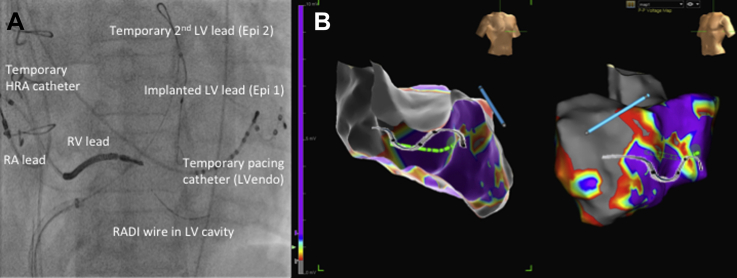

Cardiac resynchronization therapy (CRT) is a highly effective treatment for patients with heart failure, severe left ventricular (LV) impairment and a prolonged QRS duration 1, 2, 3. Despite advancing techniques a substantial proportion of individuals do not derive clinical benefit (4). Improving CRT response is particularly challenging in ischemic patients because epicardial LV lead placement within myocardial scar has a negative impact 5, 6 and avoiding scarred regions may improve CRT response (7). Furthermore, correction of electrical dyssynchrony through targeting of the latest point of activation may be important in improving CRT response 8, 9. Stimulation of the LV endocardium (LVendo), which is not constrained to the epicardial coronary venous anatomy, may provide superior hemodynamics 10, 11, 12 and improved CRT response, which may be of particular benefit in ischemic patients and nonresponders to conventional CRT 12, 13, 14. The site of optimal LVendo stimulation is highly variable in ischemic and nonischemic groups (15) with no reliable method to guide optimal LVendo lead placement. Cardiac magnetic resonance (CMR) could potentially identify the target for LV lead placement, being able to delineate scar and dyssynchrony (7). Endocardial contact mapping can demonstrate exquisite detail of endocardial activation as well as location and size of myocardial scar. Because patients with ischemic cardiomyopathy and myocardial scar have the poorest response to CRT and the most to gain from LVendo pacing, advanced imaging and mapping modalities may be able to guide the optimal site for endocardial LV lead delivery.

We hypothesized that in a group of ischemic patients (with demonstrable myocardial scar on CMR) and a high prevalence of CRT nonresponse, LVendo pacing would produce a superior hemodynamic response compared with the optimal epicardial response (LVepi). Furthermore, by pacing multiple sites, we sought to investigate whether the optimal site of LV stimulation (both epicardially and endocardially) could be predicted on the basis of scar and/or the latest point of electrical activation. By comparing endocardial contact mapping data with CMR, we further sought to elucidate the mechanisms of improved response with LVendo pacing and whether these imaging modalities could be used to guide the optimal LVendo pacing sites.

## Methods

The study complied with the Declaration of Helsinki and the protocol was approved by the local ethics committee. Informed consent was obtained from each patient. Patients with ischemic cardiomyopathy (judged by significant coronary artery disease and myocardial fibrosis on CMR), QRS duration of <150 ms, and previously implanted CRT (mean duration of implant 26 ± 21 months) have a phenotype of suboptimal response to CRT and were intentionally selected for study (3). Baseline assessment before CRT implant included clinical assessment (New York Heart Association functional class), 12-lead electrocardiogram, and 2-dimensional echocardiography. Patients underwent an extensive endocardial mapping protocol and acute hemodynamic study. CMR data were compared with contact mapping and hemodynamic data findings to compare optimal LVendo and LVepi pacing locations.

### Hemodynamic and electroanatomic study

Cases were performed under conscious sedation (mean 2.5 mg midazolam and 3 mg morphine) between March and December 2014. Patients with a mechanical aortic valve or significant peripheral vascular disease were excluded. CMR before CRT was performed with a standardized protocol including cine imaging and delayed enhancement sequences in both long and short axis views. Delayed enhancement was performed 10 to 15 min after administration of 0.2 mmol/kg Gadovist (Bayer Healthcare, Berlin, Germany) to identify myocardial fibrosis. Contact LV endocardial scar mapping was performed in sinus rhythm with a roving decapolar catheter (6-F Livewire medium sweep 115 cm; St Jude Medical, Sylmar, California) via femoral arterial access with a retrograde aortic approach and displayed using EnSite Velocity NavX system (St Jude Medical, Inc., St Paul, Minnesota). Multiple contact points were taken to create the endocardial geometry (mean 328 ± 133) with particular focus of delineating areas of scar. Points with a sensed bipolar electrogram amplitude of <0.5 mV were defined as scar and colored grey, points >1.5 mV were defined as representing healthy tissue and colored purple and those points in between defined as border zone with a color range (16).

**Figure 1:**
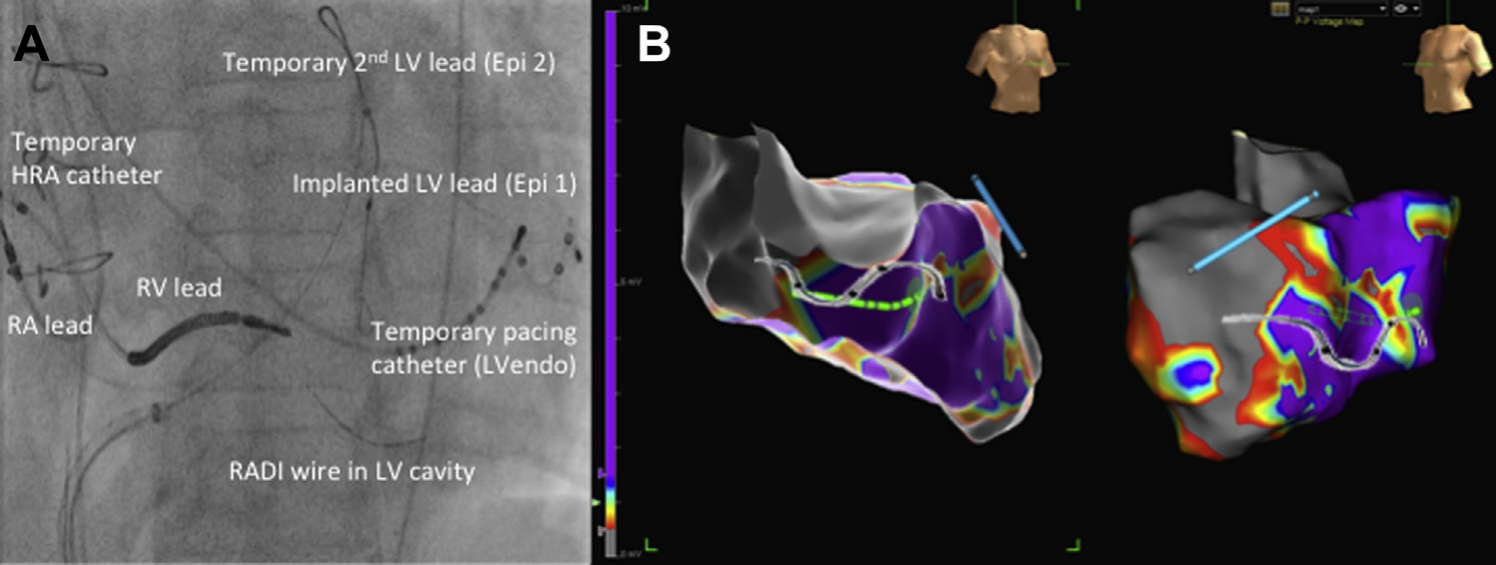
Fluoroscopic and Electroanatomic Imaging of the Study Protocol **(A)** Fluoroscopic image of the invasive protocol. **(B)** Corresponding electroanatomic endocardial, contact scar map using a decapolar left ventricular (LV) catheter and the EnSite Velocity NavX system (St. Jude Medical, Inc., St. Paul, Minnesota); right anterior oblique **(left)** and left anterior oblique **(right)** projections. Data points with a sensed electrogram amplitude of <0.5 mV were defined as scar **(grey)**, those with voltage of >1.5 mV were defined as healthy tissue **(purple)** and those points in between were in the scar border zone with a color range. The anterior surface of the heart in the **left panel** has been removed to see the location of the endocardial catheter **(green)** and distal tip **(green circle)**. The epicardial pacing (LVepi)2 lead is in an anterior vein and displayed in **blue** on the EAM. In addition, the position of the implanted (LVepi)1 lead is shown on fluoroscopy and has been superimposed on the electroanatomic map in both views. Epi = epicardial pacing; HRA = high right atrial; LVEndo = endocardial pacing; RA = right atrial; RADI = LV pressure wire; RV = right ventricular.

Temporary placement of a high right atrial quadripolar catheter was used for atrial sensing. Initially, the optimal epicardial site with atrial synchronous biventricular pacing was assessed using the patient’s chronically implanted LV lead (LVepi1) and a second, temporary epicardial LV lead placed via the femoral vein (LVepi2) to allow multiple epicardial pacing sites from different veins and along the same vein (Figure 1). The optimal endocardial site was then assessed using the roving LV endocardial decapolar catheter. The LV was divided into 12 locations (anterior, lateral, inferior, septal at basal, mid and apical levels) and randomized (Microsoft Excel). We planned to pace in each of these 12 regions per patient; in addition, extra positions in or adjacent to scar on the EAM were obtained. The mean number of endocardial positions was 10.4 ± 4.8 (range 4 to 18) and each patient had a minimum dataset comprising an anterior, lateral, inferior, and septal position. In all patients, we performed biventricular pacing at corresponding positions endocardially and epicardially at 2 sites (i.e., LVendo pacing opposite both epi1 and epi2). We used fluoroscopy (both left anterior oblique and right anterior oblique views) to show the position of the decapolar catheter in relation to the implanted leads and corroborated this with the 3-dimensional geometric shell on EAM to confirm its anatomic position with respect to an AHA 16-segment model. We confirmed the decapolar catheter tip was in a stable, fixed position before commencing our pacing protocol. Prior experience with this equipment has demonstrated stable electrode localization with mean change in position of 0.2 ± 1.7 mm (x axis), 0.1 ± 0.3 mm (y axis), and 0.2 ± 0.6 mm (z axis) (17).

The acute hemodynamic response (AHR) was measured using an 0.014-inch high-fidelity Certus RADI PressureWire in the LV as previously described (11). Atrial pacing 10 bpm greater than the intrinsic rate was used as baseline and compared with conventional epicardial biventricular pacing via the implanted LV lead (LVepi1), the temporary LV lead (LVepi2), and biventricular endocardial pacing (LVendo) at multiple sites. A delay of 20 s was respected after changing the pacing protocol and before any measurement to allow for a steady state 18, 19. Points either side of an ectopic beat were removed manually after each case. Atrioventricular delays were fixed at 100 ms and ventriculoventricular delay was set to 0 ms (simultaneous stimulation from both right and left ventricular lead poles). For each pacing site and endocardial position, we measured LV dP/dt_max_ (AHR, mm Hg/s), sensed LV electrogram amplitude (mV), sensed electrical delay (first ventricular depolarization [Q-LV]) in sinus rhythm (ms), stimulation-QRS onset (ms), and paced QRS duration (ms). Default band pass filter settings of 30 to 300 Hz were used to measure a sensed, bipolar peak-to-peak voltage signal for the temporary epicardial LV lead (LVepi2) and endocardial catheter (LVendo) on the EnSite Velocity NavX system. The sensed LV signal from the implanted (LVepi1) lead was obtained through a printed recording from the pacing systems analyzer with manual calculation of the peak-to-peak signal. We identified whether pacing locations were in or adjacent to myocardial scar identified on the EAM and CMR and measured the distance between the LV tip and the central scar zone for each data point. Field scaling was applied to account for impedance anomalies and to ensure a 1:1 representation of the patient’s cardiac geometry (EnSite Velocity NavX system). An AHR was deemed positive at a pacing location if the dP/dt_max_ increased by >10% compared with baseline measurements (20).

### Statistical methods

Continuous variables with a Gaussian distribution were described using mean ± SD. Categorical data were described by an absolute number of occurrences and associated frequency (%). Acute hemodynamic and electrical data passed the Shapiro-Wilk test for normality. To account for the clustering of data and multiple measurements within each patient, a mixed effect model was applied for all data points that achieved capture. For the best and worst achievable AHR per patient, in addition to endocardial opposite epicardial AHR data, mean differences between epicardial and endocardial datasets were compared with a 2-tailed, paired *t* test. Analysis of variance was performed to compare the means of several groups. Results were considered significant at p < 0.05. Analysis was performed on PASW Statistics 22 (SPSS Inc., Chicago, Illinois) and Stata (Stata Corp., College Station, Texas).

## Results

Mean native QRS duration was 140 ± 7 ms on surface ECG. Seven patients (88%) were labelled as having left bundle branch block; however, only 3 (38%) fulfilled the stricter Strauss criteria (21). All patients, however, had evidence of myocardial fibrosis on CMR. Patient demographics are shown in Table 1. Procedural time was 160 ± 45 min and there were no procedural complications. Patients had severe LV dysfunction with a mean ejection fraction of 27%, with minimal improvement at 6 months (ejection fraction 29%). A total of 135 epicardial and endocardial pacing sites were tested in 8 patients, 119 of which produced capture allowing electrical and hemodynamic assessment. The remaining 16 data points did not capture the ventricle at maximal (10 V) output and were located in regions of scar on the contact map. The mean number of epicardial pacing sites was 5.8 ± 0.5 and the mean number of LVendo positions was 10.4 ± 4.8, including sites where LV capture was unsuccessful.

### Acute hemodynamic response

**Figure 2:**
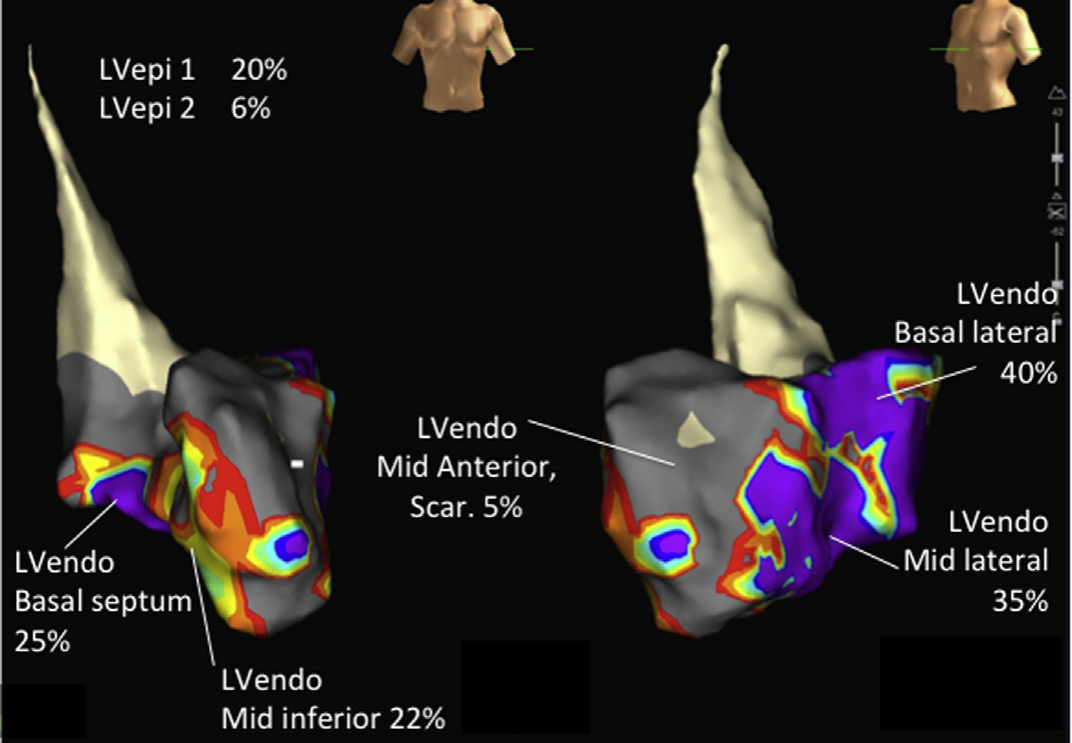
Electroanatomic Contact Scar Map With Associated Acute Hemodynamic Responses During Biventricular Pacing at Different Sites Anteroposterior **(left)** and left anterior oblique **(right)** projections. Data points with a sensed electrogram amplitude of <0.5 mV were defined as scar **(grey)**, those with voltage >1.5 mV were defined as healthy tissue **(purple)**, and those points in between were in the scar border zone with a color range. The best epicardial (LVepi1 and LVepi2) acute hemodynamic response (% change in dP/dt, mm Hg compared with baseline during biventricular pacing) is displayed alongside 5 endocardial (LVendo) positions. Abbreviations as in Figure 1.

Using a mixed effect model analysis for all data points, the mean AHR of LVendo positions (11.81% [range -7.2% to 44.6%]) was significantly superior to the mean AHR of LVepi positions (6.55% [range -11.0% to 19.7%]; p = 0.025) (Tables 2 and 3, Online Table 1, Figure 2). This was associated with an identical Q-LV between groups (LVendo 75 ms [range 13 to 161 ms] vs. LVepi 75 ms [range 25 to 129 ms]; p = 0.354) but a shorter stimulation-QRS duration (LVendo 15 ms [range 7 to 43 ms] vs. LVepi 19 ms [range 5 to 66 ms]; p = 0.010) and paced QRS duration (LVendo 149 ms [range 95 to 218 ms] vs. LVepi 171 ms [range 120 to 235 ms]; p < 0.001). The mean of the best achievable AHR at the optimal LVendo site was significantly higher than the optimal LVepi pacing site (25.64 ± 14.74% vs. 12.64 ± 6.76%; p = 0.044). This was associated with a trend toward a longer Q-LV (95 ± 38 ms vs. 67 ± 33 ms; p = 0.216), shorter stimulation-QRS duration (14 ± 5 ms vs. 19 ± 8 ms; p = 0.126) and a shorter paced QRS duration (137 ± 26 ms vs. 167 ± 33 ms; p = 0.002). The difference between the best versus worst LVepi site was highly significant (p = 0.0014) as it was for best versus worst LVendo (p = 0.0002). There was a greater difference between the best and worst AHR with LVendo pacing compared with LVepi pacing 26.57 ± 12.58 vs. 13.44 ± 9.00; p = 0.03. There was a nonsignificant trend toward an improved AHR with basal compared with mid and apical positions at all sites (LVepi and LVendo) (base 13.7 ± 11, mid 8.1 ± 9.8, apex 9.6 ± 12.7; p = 0.07).

### Optimal LV site location

**Figure 3:**
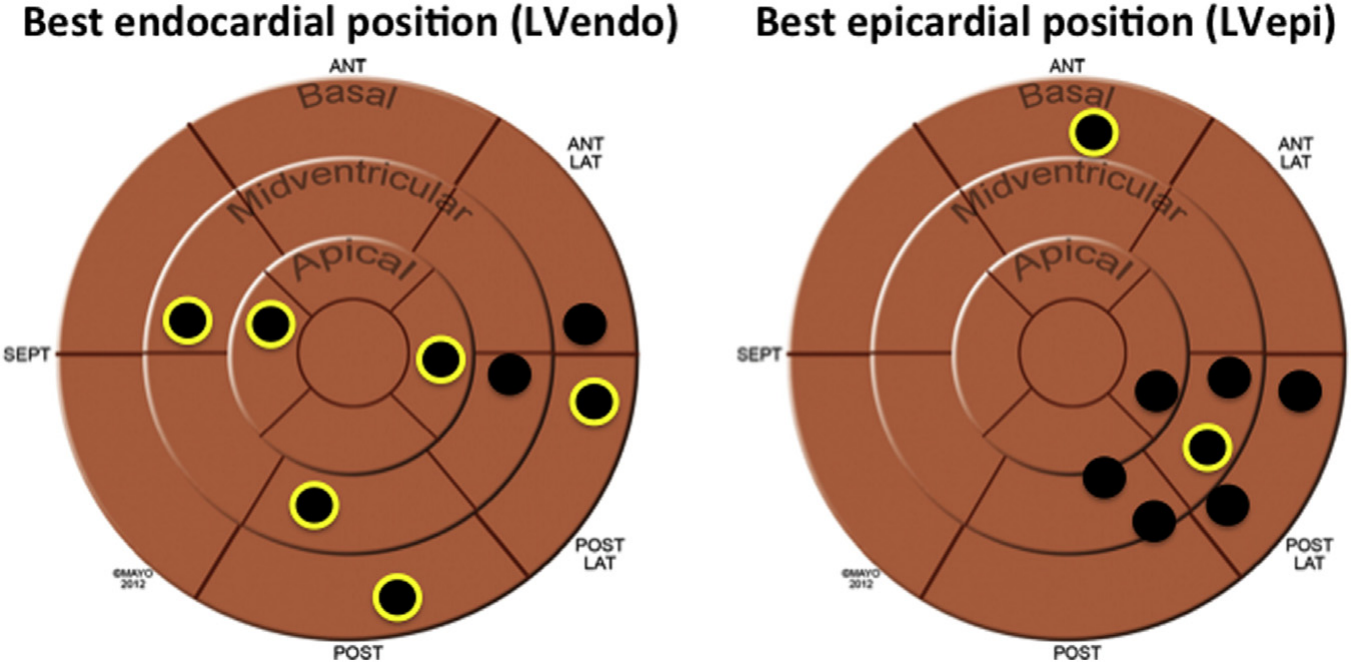
The Optimal Site for LV Stimulation During Biventricular Pacing Optimal endocardial **(left)** and epicardial **(right)** sites (by acute hemodynamic response [AHR]) for placement of the LV lead in the 8 patients. Black circles with a yellow circumference represent the best overall location (LVendo vs. LVepi). This demonstrates that in 6 patients, LVendo pacing produced the best AHR and the optimal locations were dispersed throughout the geometry of the LVendo. Two patients had the best AHRs achieved with LVepi pacing; as can be seen the pacing locations were clustered due to the constraints of the epicardial veins. AHA = American Heart Association; ANT = anterior; ANT LAT = anterior lateral; POST = posterior; POST LAT = posterior lateral; SEPT = septal; other abbreviations as in Figure 1.

Epicardial stimulation sites were limited according to the coronary venous anatomy and the best achievable epicardial pacing locations were therefore confined to the AHA segments subtended by these veins. In 7 of 8 patients, the optimal LVepi pacing site was inferolateral/inferior; in the other patient the basal anterior site was best in keeping with the belief that pacing from the inferolateral/inferior wall is the optimal site of epicardial stimulation in the majority of subjects. LVendo pacing was not limited to the distribution of the coronary veins and there was substantial individual variation in the optimal site producing the best AHR (Figure 3).

### Endocardial pacing opposite epicardial lead

When the endocardial catheter was placed opposite the pacing tip of the epicardial lead on the basis of screening and EAM (Figure 1) (LVepi1/posterolateral and LVepi2/anterior vein), the AHR was significantly greater (15.2 ± 10.7% vs. 7.6 ± 6.3%; p = 0.014) despite a similar Q-LV (LVendo 70 ± 38 ms vs. LVepi 79 ± 34 ms; p = 0.512); however, the paced QRS duration was significantly shorter with LV endocardial pacing (LVEndo 137 ± 22 ms vs. LVepi 166 ± 30 ms; p < 0.001).

### Pacing in scar and late activated sites

The voltage amplitude of the LV sensed electrogram signal was lower at LVendo compared with LVepi sites (LVendo 3.84 ± 2.9 mV vs. LVepi 6.68 ± 4.6 mV; p = 0.002). Of the LVendo stimulation sites, 16 did not capture and were in scar at a maximal output of 10 V (confirmed by voltage contact mapping and CMR). The mean electrogram amplitude within scar sites with noncapture was 0.18 ± 0.12 mV (n = 16). The optimal hemodynamic LVendo site was not at the site of latest electrical activation on the EAM in 6 of 8 patients (Online Table 1).

## Discussion

We studied the optimal site for both LVepi and LVendo stimulation in a cohort of patients with ischemic heart disease with poor response to conventional CRT. The principal findings were as follows.

1. Indiscriminate endocardial pacing was superior to epicardial stimulation, associated with a similar first ventricular depolarization, shortened stimulation-QRS duration and shortened paced QRS duration.
2. Optimal achievable endocardial AHR was superior to the optimal achievable epicardial AHR.
3. There was significant interindividual variability of the position of the optimal LVendo site which was not predicted by the site of latest electrical activation on EAM.
4. Pacing within scar on EAM and CMR (both epicardial and endocardial positions) resulted in failure to capture and a poor AHR.
5. LVendo stimulation at a site approximating LVepi stimulation resulted in a better AHR and shorter paced QRS duration despite a similar Q-LV.

### Comparison with previous studies

In keeping with the current study, LVendo pacing has been found to be superior to conventional CRT in both ischemic and nonischemic patients 11, 12, 13. Derval et al. (22) found that LVendo pacing was superior to posterolateral LVepi pacing in nonischemic patients with significant individual variation between the optimal LVendo pacing sites (22). Spragg et al. (10) undertook EAM and AHR measurement in 11 patients with ischemic heart disease and dyssynchronous heart failure at the time of ventricular tachycardia ablation and found LVendo was superior to LVepi pacing (10). Our group has shown the superiority of LVendo pacing over conventional CRT in a group of ischemic and nonischemic patients (11). The superiority of LVendo pacing over LVepi seems to be reproducible, but with a significant variability in the optimal site between patients in all the aforementioned studies 10, 11, 12.

The current study has some important differences and new findings in comparison with previous studies that may be of clinical importance. First, our subjects were selected on the basis of having factors associated with a suboptimal response to conventional CRT, namely, male sex, ischemic cardiomyopathy, myocardial fibrosis (on CMR), and a QRS duration of 120 to 150 ms. Although all patients were ischemic in the study by Spragg et al. (10), our subjects had a narrower QRS duration (140 ± 14.9 ms vs. 176 ± 29 ms). In the prior studies, epicardial biventricular pacing was only delivered from a single site in the posterolateral vein. The superiority of LVendo in these studies 10, 11, 12 may have, therefore, arisen because the optimal LVepi position was not assessed. In the current study, we rigorously performed epicardial pacing from multiple sites (between and along veins) to first determine the optimal LVepi site (mean 5.8 ± 0.5 sites per subject). For the first time our findings confirm the superiority of LVendo stimulation, when both LVendo and LVepi sites are systematically optimized. Figure 2 shows an example of the superiority of LVendo over LVepi pacing even when LVepi pacing produced a good AHR.

**Figure 4:**
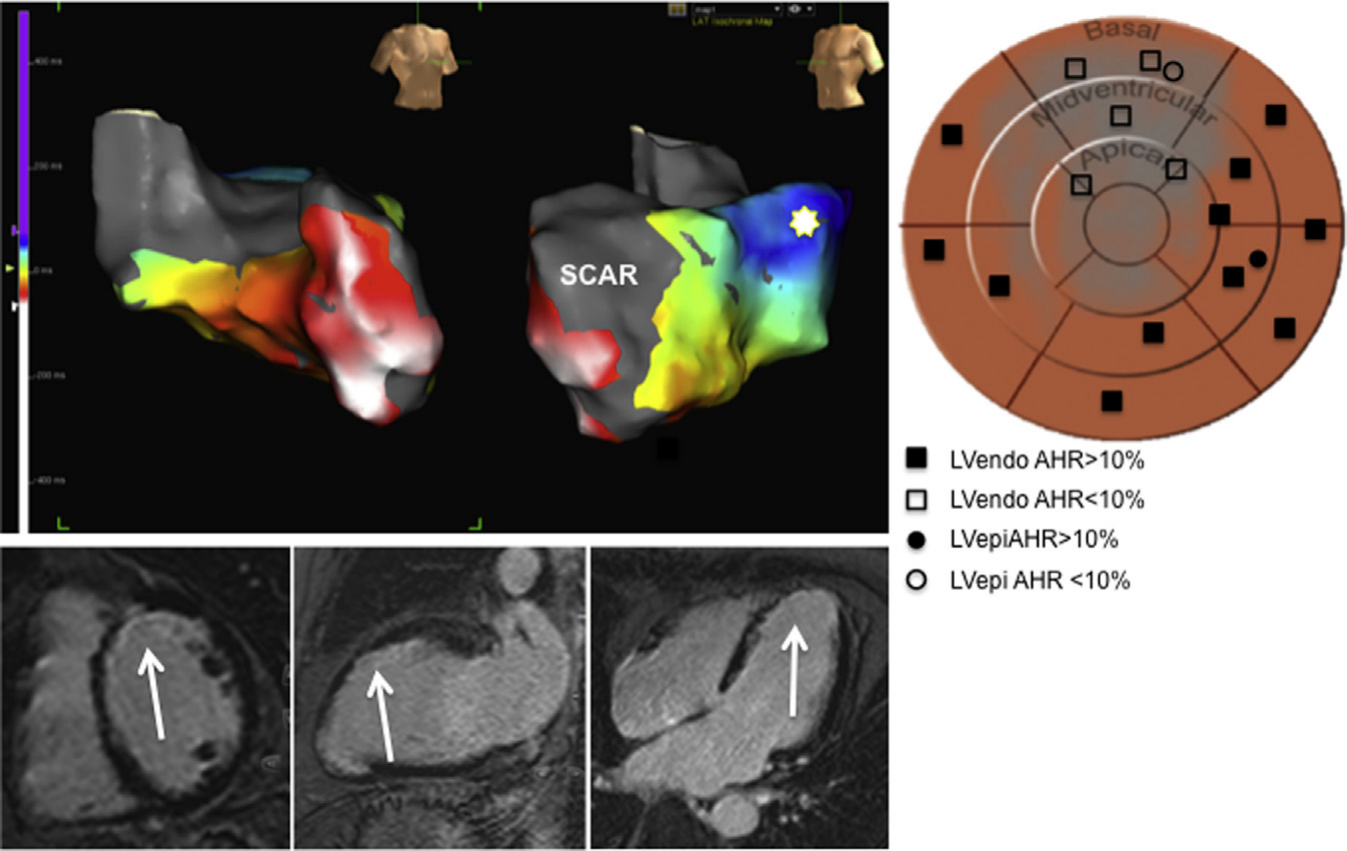
Local Activation Map, Correlation With Myocardial Fibrosis on CMR and Associated AHR at Different Locations in 1 Patient **(Top)** Electroanatomic (EAM) contact map showing local activation in the same subject as in Figure 2. **White** signifies earliest activation and **blue** latest activation, demonstrating the basal lateral region as the site of latest electrical delay. In this case, the optimal AHR **(star)** matched the site of latest electrical delay, which was distant from ischemic scar. **(Bottom)** Cardiac MR (CMR), late gadolinium enhancement sequences in the short axis, mid ventricular **(left)**, 2-chamber **(middle)**, and 4 chamber **(right)** views. The **white arrows** demonstrate areas of thin walled myocardium with associated subendocardial myocardial fibrosis, corresponding to an left anterior descending (LAD) territory myocardial infarction. There is a close correlation between the scar demonstrated on the EAM and that displayed with CMR. **(Right)** AHA bulls-eye plot diagram with scar (derived from CMR and EAM) spray painted in **grey** (anterior, LAD infarct). All different positions for the LV lead are demonstrated (both epicardial and endocardial) with the legend detailing whether the associated AHR with biventricular pacing was <10% or >10% improvement from baseline. Pacing around the anterior regions of scar corresponded to a poor AHR, compared with much better AHRs in sites out of scar. Abbreviations as in Figures 1 and 3.

A notable finding of the current study was the superiority of LVendo over LVepi at the same site. Previous studies have suggested no difference in ischemic and nonischemic patients, however we found a significant benefit with endocardial pacing (15.2 ± 10.7% vs. 7.6 ± 6.3%; p = 0.014). Spragg et al. (10) compared LVendo and LVepi at the same site in 7 patients and found LVendo pacing increased dP/dt_max_ by 36% from baseline compared with 29% via LVepi although this increase was not statistically significant. These differences may be related to the different population studied, but also that we assessed and optimized LVepi pacing in both the anterior and posterolateral regions. Our data are in keeping with both animal studies (13) and a computer modelling study from our group (14). Finally, our patients had CMR data that could be correlated with the results of EAM, which was not the case in the aforementioned studies (Figure 4).

### Scar and electrical activation

**Figure 5:**
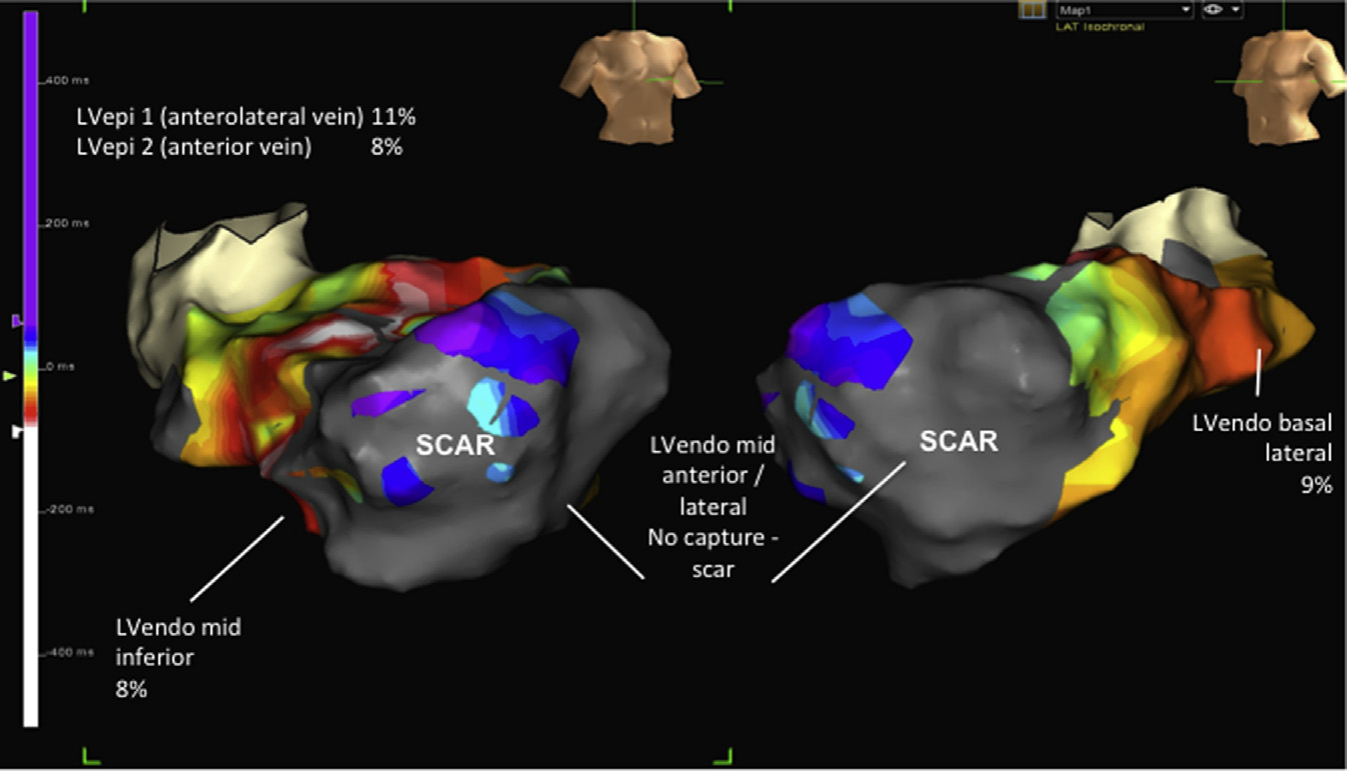
Local Activation Map and Associated Acute Hemodynamic Response in a Patient With Electrical Latency Within a Large Area of Scar Dilated, globular heart with a heavy burden of myocardial scar. Earliest activation is **white** and latest activation **blue/purple**. In this case, LVendo locations were not superior to conventional LVepi with respect to the AHR. The point of latest electrical activation in this case is around the anteroseptum, most likely as a result of slow activation spreading and encircling a large region of scar. Although these sites are the latest activated they will not produce a good AHR because they are in scar and may explain why the latest activated site is not always the optimal pacing site. Abbreviations as in Figures 1 and 3.

In the study from Spragg et al. (10), optimal LVendo pacing sites typically were located far from regions of dense scar and in 8 of 11 subjects optimal LVendo sites were not at the site of latest activation. For most patients, optimal pacing sites were located in regions activated neither extremely early nor late during ventricular excitation. The authors concluded the lack of correlation between latest endocardial activation sites and optimal pacing sites may reflect a disconnect between electrical and mechanical activation, the impact of regions of slow conduction, and lines of conduction block on optimal pacing sites. Our results support this theory, confirmed by failure to capture the LV at maximal voltage at 16 endocardial sites within scar (on CMR and EAM). Furthermore, the optimal hemodynamic LVendo site did not necessarily correlate with the site of latest electrical activation in 6 of 8 patients, as determined on the EAM activation map. In keeping with this finding, the Q-LV at the optimal LVendo sites were not significantly longer than those at the optimal LVepi site. It seems likely that, on the basis of our and other prior findings, the latest activating site is not necessarily the optimal site to pace with respect to improved hemodynamics. This may be due to localized areas of slow conduction with islands of viable tissue within areas of scar that activate late (and therefore have a long Q-LV). Likewise, when stimulation is performed at that site, impulse propagation is also slow out of this area and does not result in effective resynchronization. An example of this is seen in Figure 5 in a patient with a large circumferential midventricular and apical infarct with areas of late activating tissue within the scar. It is possible that seeking a late activated site may be beneficial, but only if conduction out of that site is not also delayed or blocked by regions of scar. This is analogous to a Goldilocks effect where the optimal site in ischemic patients because of scar/slow conduction may be not too early, not too late but just right, somewhere in between the two.

### Mechanism of benefit of LVendo pacing

Indiscriminate stimulation of the LVendo produced a superior hemodynamic effect compared with conventional epicardial stimulation. The benefit of LVendo pacing may be related to the lack of coronary venous constraints and the ability to access all regions of the myocardium resulting in the ability to obtain the best achievable AHR. The broad variation in hemodynamic response and notable lack of capture in areas of scar, clearly visible on CMR/EAM suggest a targeted approach avoiding scar may be helpful. The superiority of the optimal LVendo over the optimal LVepi AHR at the same site with an associated shorter paced QRS duration may support more rapid activation of the ventricles by fast conducting tissue including the His-Purkinje network 14, 15. Equally, other mechanisms (not examined in this manuscript) such as a shorter, more concave path for electrical conduction with LVendo pacing may also be contributing to our findings.

### Clinical relevance

CRT nonresponse occurs in one-third of patients receiving this treatment and is higher in ischemic patients with myocardial fibrosis and modest QRS prolongation (120 to 150 ms). Strategies to improve response in this group are required. The recently published ALSYNC (ALternate Site Cardiac ResYNChronization) study demonstrated the feasibility and safety of LV endocardial CRT delivered, through the atrial trans-septal approach (23). In 138 patients with either prior suboptimal response to conventional CRT, failure of LV lead implantation or suboptimal coronary venous anatomy the investigators achieved a high implant success rate (89.4%), with stable pacing parameters and an 82.2% freedom from complications at 6 months. Furthermore, clinical and echocardiographic improvement was 59% and 55%, respectively, in a group with prior nonresponse, which is highly encouraging. Targeting the optimal site for LV stimulation remains an important issue given the marked degree of variability between patients. Our results suggest that CMR may be helpful in this respect especially in avoiding areas of scar, which result in failure to capture or poor resynchronization. Targeting a late but not necessarily latest activating site (i.e., late but not within the scar) is achievable with CMR techniques that give information regarding myocardial activation/contraction patterns as well as pinpointing scar (24).

The ALSYNC study used an empirical approach to LV lead placement and despite this reported a significant improvement compared with conventional epicardial pacing. Our results support image guidance on the basis that a broad range of AHR values were obtained and therefore not all endocardial positions are equal in this cohort. Although an indiscriminate approach showed endocardial pacing was superior to epicardial pacing (Table 2), an even greater AHR was achievable when both epicardial and endocardial sites were optimized. Therefore, an image-guided, targeted approach could be a strategy for identifying the optimal location for LV lead stimulation. CMR techniques do, however, require further evaluation to assess their merit in guiding endocardial pacing sites and techniques which allow CMR derived scar and mechanical activation to be fused onto live fluoroscopy for epicardial LV lead guidance may also be used for LVendo lead guidance 18, 25.

### Study limitations

The main limitation of the current study is the low number of patients studied. Due to the highly invasive nature of the study and the difficulty in identifying a group of nonresponders, this is understandable. The protocol was, however, extremely rigorous with a large number of data points (135 endocardial and epicardial pacing positions). The EAM study is invasive and not likely to be of use in routine clinical practice. Due to the length of the procedures no changes in atrioventricular or ventriculoventricular delay were studied and it is possible that such manipulations may have produced a different response. Finally, hemodynamic response may not necessarily translate to chronic response to CRT and therefore longer term studies are required; results from a multicenter, randomized, controlled trial, RADI-CRT (Pressure Wire Guided Cardiac Resynchronisation Therapy; NCT01464502) may help to clarify this.

## Conclusions

Our findings suggest that, in ischemic patients with poor CRT response, endocardial pacing is superior to epicardial pacing with an even greater response achievable with optimization for each set of protocols. The mechanism of benefit may be due to the ability to access more optimal sites that cannot be reached by the constraints of the CS anatomy. Furthermore, guidance to the optimal LV pacing site may be aided by modalities such as CMR to target nonscarred and delayed activating sites.

Perspectives **COMPETENCY IN MEDICAL KNOWLEDGE:** Biventricular pacing delivered through LV endocardial stimulation seems to provide superior acute hemodynamics and a shortening of the paced QRS duration compared with LV epicardial stimulation in the equivalent territory. A lack of coronary venous constraint and preferential access to fast conducting tissue are likely to under pin the superiority of this method for delivery of CRT. Cardiac magnetic resonance–derived myocardial fibrosis corroborates with scar derived from electroanatomic maps and may be helpful in guiding LV lead placement. Furthermore, targeting nonscarred segments identified through cardiac magnetic resonance cine sequences can aid optimal lead placement in an attempt to improve response to CRT.

**TRANSLATIONAL OUTLOOK:** Biventricular pacing has been offered to selected patients with heart failure for more than 20 years; however, a significant proportion of individuals receiving this therapy do not derive significant clinical benefit. Certain groups such as male patients with ischemic etiology, myocardial scar and QRS duration of 120 to 150 ms have a high prevalence for poor response and alternative options for improving the effectiveness of CRT are urgently required. Recently, animal and human data have suggested superior hemodynamics and shorter electrical activation for endocardial versus epicardial LV stimulation in delivery of biventricular pacing. Clinical studies have demonstrated the feasibility of endocardial LV stimulation through transatrial septal and transventricular septal approaches, as well as leadless LV pacing in combination with an implanted right-sided system. Coupled with advanced cardiac imaging to guide LV placement away from areas of scar and toward those activating lately, CRT delivery through permanent LV endocardial stimulation may represent a new method to improve outcomes for heart failure patients over the coming decades.

## Figures and Tables

**Table 1:** Demographic Data, Pre-CRT and Post-CRT Outcomes (N = 8)

Age (yrs)	71 ± 7.4
Male (%)	8 (100)
LVEF by 2D echocardiography Simpson’s biplane before CRT	27 ± 7.4
SDI derived from echocardiography (%)	19
NYHA functional class II/III, before CRT implantation	2/6
Ischemic etiology	8 (100)
Sinus rhythm	8 (100)
QRS duration (m)	140 ± 7
LBBB	7 (88)
LBBB by revised Strauss criteria	3 (38)
Echo responders at 6 months∗	2 (25)
LVEF by 2D echo, Simpson’s biplane after CRT	29 ± 7.9
Clinical responders at 6 months†	3 (38)

Values are mean ± SD or n (%).

2D = 2-dimensional; CRT = cardiac resynchronization therapy; LBBB = left bundle branch block; LVEF = left ventricular ejection fraction; NYHA = New York Heart Association.

∗ Echocardiographic response using 2D transthoracic echocardiography; confirmed if ≥15% reduction in end-systolic volume (ESV) at 6 months’ follow-up compared with before implantation.

† Clinical response to CRT using Packer’s clinical composite score (26).

**Table 2:** Mixed Effect Model

	Mean Difference	95% Confidence Interval	p Value
Change in AHR (%)	4.23	0.52 to 7.93	0.025
QLV (ms)	−5.92	−18.45 to 6.60	0.354
Stimulation-QRS duration (ms)	−3.70	−6.50 to 0.88	0.010
Paced QRS duration (ms)	−25.45	−33.59 to −17.32	<0.001

Mixed effect model for all data points achieving capture comparing epicardial and endocardial pacing across the dependent variables as shown. A total of 32 epicardial and 87 endocardial data points were compared across 8 patients.

AHR = acute hemodynamic response; QLV = first ventricular depolarization (earliest onset QRS duration on surface 12 lead electrocardiogram) to the nadir signal on the LV lead electrogram.

**Table 3:** Mean Differences Between Epicardial and Endocardial Datasets

	Epicardial	Endocardial	p Value
Best achievable AHRs in each patient	(N = 8)	(N = 8)	
Mean change in AHR (%)	12.64 ± 6.76	25.64 ± 14.74	0.044
Mean QLV (ms)	67 ± 33	95 ± 38	0.216
Mean stimulation-QRS duration (ms)	19 ± 8	14 ± 5	0.126
Mean paced QRS duration (ms)	167 ± 33	137 ± 26	0.002
Worst AHR in each patient	(N = 8)	(N = 8)	
Mean change in AHR (%)	−0.80 ± 6.81	−0.93 ± 3.79	0.964
Mean QLV (ms)	68 ± 39	59 ± 15	0.556
Mean stimulation-QRS duration (ms)	26 ± 21	16 ± 4	0.187
Mean paced QRS duration (ms)	174 ± 32	154 ± 34	0.164
Comparison of LVendo opposite the corresponding position of LVepi1 and LVepi2	(N = 16)	(N = 16)	
Mean change in AHR (%)	7.60 ± 6.3	15.2 ± 10.7	0.014
Mean QLV (ms)	79 ± 34	70 ± 38	0.512
Mean stimulation-QRS duration (ms)	20 ± 13	16 ± 7	0.214
Mean paced QRS duration (ms)	166 ± 30	137 ± 22	<0.001

Values are mean ± SD.

LVendo = endocardial pacing; LVepi = epicardial pacing; other abbreviations as in Tables 1 and 2.
