# Supplementary material for: Optimized Left Ventricular Endocardial Stimulation Is Superior to Optimized Epicardial Stimulation in Ischemic Patients With Poor Response to Cardiac Resynchronization Therapy: A Combined Magnetic Resonance Imaging, Electroanatomic Contact Mapping, and Hemodynamic Study to Target Endocardial Lead Placement
Source: JACC Clin Electrophysiol. 2016 Dec;2(7):799–809. doi: 10.1016/j.jacep.2016.04.006 (PMC5196018; doi:10.1016/j.jacep.2016.04.006)
Supplement: Online Table 1 [file mmc1.docx]

**ONLINE TABLE**

| Patient | QRSd (ms) | Morphology | Subendocardial scar location | Latest electrical delay | Optimal LV site | Best Epicardial AHR (%) | Best Endocardial AHR (%) |
| --- | --- | --- | --- | --- | --- | --- | --- |
| 1 | 136 | IVCD | Anterior | Basal Lateral | Mid Postero-Lateral (Epi) | 14.1 | 12.2 |
| 2 | 140 | LBBB | Anterior | Basal Lateral | Basal Lateral (Endo) | 19.7 | 39.6 |
| 3 | 142 | LBBB | Antero-Septal | Basal Lateral | Basal Inferior (Endo) | 5.6 | 44.6 |
| 4 | 148 | LBBB | Antero-Lateral | Basal Lateral | Apical Septal (Endo) | 10 | 43.1 |
| 5 | 132 | IVCD | Anterior | Basal Antero-Lateral | Mid Inferior (Endo) | 12 | 14 |
| 6 | 144 | IVCD | Septal | Basal Lateral | Mid Septal (Endo) | 23 | 25.6 |
| 7 | 130 | IVCD | Septal,  Antero-Lateral | Basal Lateral | Apical Lateral (Endo) | 5.3 | 16.6 |
| 8 | 146 | IVCD | Circumferential | Mid Antero-Septal | Basal Anterior (Epi) | 11.4 | 9.4 |

Summary table for the 8 patients studied. Morphology denotes surface ECG prior to initial CRT insertion; Left bundle branch block (LBBB), Intraventricular conduction delay (IVCD). Subendocardial myocardial fibrosis region is as described on CMR late gadolinium enhancement sequences. Latest electrical delay refers to the propagation map from EAM during intrinsic rhythm during the pacing study. Optimal site refers to the location of the LV lead (pacing tip poles) which produced the greatest AHR during biventricular pacing; epicardial (Epi) or endocardial (Endo). The best AHR with biventricular epicardial and endocardial pacing is shown per patient.
